# Mediastinal esophageal leiomyosarcoma abutting a retroesophageal right subclavian artery: A case report

**DOI:** 10.1016/j.ijscr.2019.06.052

**Published:** 2019-06-26

**Authors:** Erin M. Corsini, Daniel Maoz-Metzl, Kyle G. Mitchell, Robert D. Rice, Boris Sepesi

**Affiliations:** a Department of Thoracic and Cardiovascular Surgery, MD Anderson Cancer Center, Houston, TX, United States; b Division of Cardiothoracic Surgery, Department of Surgery, University of New Mexico, Albequerque, NM, United States; c Department of Thoracic and Cardiovascular Surgery, Dwight D. Eisenhower Army Medical Center, Fort Gordon, GA, United States

**Keywords:** Esophageal leiomyosarcoma, Retroesophageal subclavian artery, Arteria lusora, Case report

## Abstract

**Introduction:**

Esophageal leiomyosarcoma and arteria lusoria represent rare entities in thoracic disease. In the setting of arteria lusoria, careful surgical planning and preoperative imaging is essential.

**Presentation of case:**

A 53-year old male presented with a history of cough. Imaging demonstrated a 7-cm mediastinal mass at the level of the trachea and upper esophagus, abutting an incidentally found retroesophageal right subclavian artery. The right carotid artery originated from the aortic arch.

Comprehensive evaluation demonstrated a mediastinal sarcoma. Given the tumor size and close association with vital structures, induction chemotherapy was given without response. He was therefore taken for resection, including possible esophagectomy. The mass was well-encapsulated, permitting mobilization off the surrounding structures, and was identified arising from the muscular layer of the esophagus. Postoperatively, he had a low-volume chyle leak, which resolved with conservative management. He was offered mediastinal adjuvant radiation due to close margins, but declined. The patient remained without evidence of disease for over two years, at which time a single pulmonary metastasis was noted and resected.

**Discussion:**

Esophageal leiomyosarcoma are an uncommon intrathoracic malignancy and may present with symptoms of mass effect. While patients with arteria lusoria are typically asymptomatic, they may suffer surgical morbidity if this entity is not identified preoperatively. In this case, the aberrant artery was intimately involved with the leiomyosarcoma, increasing complexity and rendering complete oncologic resection challenging.

**Conclusion:**

Herein we present a challenging surgical resection of esophageal leiomyosarcoma in the setting of arteria lusoria, which was successfully treated tumor enucleation.

## Highlights

• Esophageal leiomyosarcoma represent a rare esophageal malignancy.
• Arteria lusoria is defined by the anomalous development of an aberrant right subclavian artery posterior to the esophagus.
• Comprehensive and detailed preoperative planning were essential to a successful esophagus-preserving resection.

## 1 Introduction

**Fig. 1:**
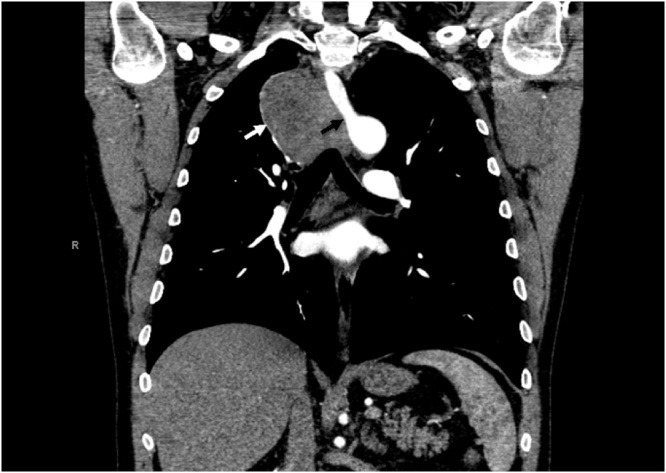
CT demonstrating retroesophageal SCA (black arrow) originating from aorta, medial to esophageal leiomyosarcoma (white arrow).

**Fig. 2:**
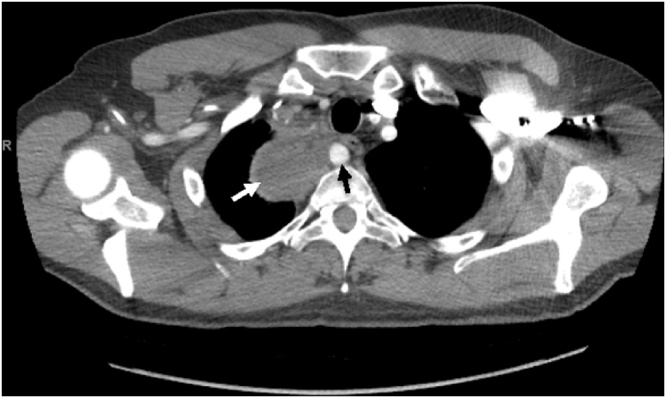
CT demonstrating retroesophageal SCA (black arrow) adjacent to esophageal leiomyosarcoma (white arrow).

Esophageal leiomyosarcoma is a rarely reported smooth muscle esophageal tumor characterized by sheets of spindle-shaped cells arranged in fascicles with associated increased number of mitotic figures [1,2]. Arteria lusoria represents the congenital anomalous development of an aberrant right subclavian artery (SCA),^1^ resulting in posterior coursing of the SCA behind the esophagus, and is likewise rare. The vessel originates from either the brachiocephalic artery or aorta directly [3]. This work has been reported in line with the SCARE criteria [4].

## 2 Case presentation

A healthy 53 year-old male presented with a 6-month history of non-productive cough. He had no significant past medical or surgical history and was a nonsmoker. Computed tomography (CT) demonstrated 7.4 × 5.2-cm right posterior mediastinal mass, abutting the trachea, superior vena cava, ascending aorta, and esophagus, as well as an incidentally noted retroesophageal SCA with a separate right carotid artery originating from the aortic arch (Fig. 1, Fig. 2). CT-guided percutaneous biopsy revealed high-grade leiomyosaroma. Esophagoscopy did not demonstrate esophageal mucosal involvement. Metastatic workup was negative. After multidisciplinary discussion, four cycles of neoadjuvant chemotherapy with doxorubicin and dacarbazine were given in the hope that the tumor would decrease in size, permitting wider resection margins; however, there was no objective radiographic response.

He was subsequently taken to the operating room for resection and possible esophagectomy with planned substernal reconstruction and possible ligation of the right subclavian artery. Through a fourth intercostal muscle-sparing posterolateral thoracotomy, the large mediastinal mass was noted. Because the tumor was abutting the superior vena cava, ascending aorta, retroesophageal SCA, and trachea, it was not feasible to achieve wide margins without disrupting the vital structures. However, the mass encapsulated and was able to be dissected away from the surrounding vital structures. Extra care was taken not to injure the right subclavian artery, which was skeletonized away from the tumor. The mass was arising from the proximal esophageal muscular layer. Because there was no direct invasion of surrounding tissues, the mass was resected with partial resection of the esophageal muscular layer without violation of the mucosa or tumor capsule. A previously harvested intercostal muscle flap reinforced the exposed but intact mucosal layer of the esophagus.

**Fig. 3:**
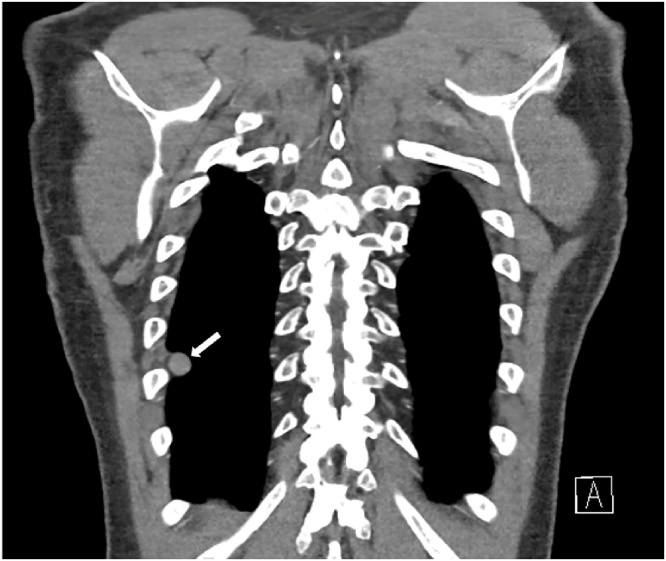
CT demonstrating isolated right pleural metastasis (white arrow).

The postoperative course was complicated by low-volume chyle leak, which resolved with conservative management. Adjuvant radiotherapy was offered, but he declined. The patient remained without evidence of disease for two years, until he was found to have a right 1.5-cm pleural-based metastasis, which was also resected (Fig. 3). He has no evidence of disease currently, now 30 months after resection of metastasis.

## 3 Discussion

Esophageal leiomyosarcomas commonly present with progressive dysphagia secondary to intraluminal compression, though other symptoms may be present including odynophagia, retrosternal chest pain, weight loss, hoarseness, cough, and emesis [1]. Barium esophogram was classically used, demonstrating a smooth intraluminal mass and sinus tract to the cavitary mass [1,2,5]. Newer imaging modalities have further aided in the preoperative characterization of these tumors [5]. Furthermore, endoscopic ultrasound-guided fine needle aspiration may provide an accurate method for preoperative histological characterization of these masses to direct therapeutic needs. Curative resection is successful in 65% of patients with 5 and 10-year survival rates of 47% and 31%, respectively [2].

In this case, though the tumor was in close relationship with the superior vena cava, ascending aorta, and retroesophageal SCA, wide surgical margins as indicated for sarcoma were not feasible. However, because the tumor was well-encapsulated and separated easily from uninvolved esophageal mucosa, an esophageal myomectomy was completed and final pathology was likely consistent with R1 resection. In the event that the subclavian artery could not have been spared, the plan was for ligation of the artery during the initial operation with staged reconstruction with carotid to subclavian bypass at a later time based upon symptoms. Vascular surgery was consulted for this possibility, and felt that the collateral blood flow may be adequate, though the need for vascular reconstruction was avoided in this instance.

Arterial lusoria represents a rare vascular anomaly. Traditionally, the right SCA develops from the right fourth congenital aortic arch [3,6]. In the case of an aberrant right SCA, the distal right fifth aortic arch persists and the artery consequently originates from the aorta distal to the left SCA, passing posterior to the esophagus and trachea on its normal rightward course [6]. This variation causes the right recurrent nerve to course directly to the larynx, rather than the usual recurrent path. Patients are generally asymptomatic, yet the displaced artery can pose definitive surgical risk, rendering preoperative anatomic definition paramount, as injury may lead to life-threatening hemorrhage [3]. In this case, the aberrant right SCA was intimately involved with the leiomyosarcoma, increasing complexity and rendering oncologic resection challenging.

This case represents a rare combination of esophageal leiomyosarcoma and aberrant right SCA. The patient was successfully treated with esophageal tumor resection while preserving the aberrant right SCA.

## Conflicts of interest

The authors have no conflicts of interest to disclose.

## Sources of funding

This research did not receive any specific grant from funding agencies in the public, commercial, or not-for-profit sectors.

## Ethical approval

MD Anderson Cancer Center Institutional Review Board – This investigation is exempt from ethical approval at our institution.

## Consent

Consent obtained.

## Author contribution

BS is the primary investigator and contributed to conceptualization, study design, and manuscript drafting and editing. DM, EC, KM, and RR contributed to data collection, data analysis, and manuscript drafting and editing.

## Registration of research studies

NA.

## Guarantor

Erin M. Corsini.

Boris Sepesi.

## Provenance and peer review

Not commissioned, externally peer-reviewed.

## Footnotes

1 SCA: subclavian artery; CT: computed tomography.
